# Quantitative Simulations Predict Treatment Strategies Against Fungal Infections in Virtual Neutropenic Patients

**DOI:** 10.3389/fimmu.2018.00667

**Published:** 2018-04-04

**Authors:** Sandra Timme, Teresa Lehnert, Maria T. E. Prauße, Kerstin Hünniger, Ines Leonhardt, Oliver Kurzai, Marc Thilo Figge

**Affiliations:** ^1^Research Group Applied Systems Biology, Leibniz Institute for Natural Product Research and Infection Biology—Hans Knöll Institute, Jena, Germany; ^2^Faculty of Biological Sciences, Friedrich Schiller University Jena, Jena, Germany; ^3^Center for Sepsis Control and Care (CSCC), Jena University Hospital, Jena, Germany; ^4^Fungal Septomics, Septomics Research Center, Leibniz Institute for Natural Product Research and Infection Biology—Hans Knöll Institute, Friedrich Schiller University, Jena, Germany; ^5^Institute for Hygiene and Microbiology, University of Würzburg, Würzburg, Germany

**Keywords:** fungal infections, neutropenia, treatment strategies, bottom-up modeling approach, computer simulations

## Abstract

The condition of neutropenia, i.e., a reduced absolute neutrophil count in blood, constitutes a major risk factor for severe infections in the affected patients. *Candida albicans* and *Candida glabrata* are opportunistic pathogens and the most prevalent fungal species in the human microbiota. In immunocompromised patients, they can become pathogenic and cause infections with high mortality rates. In this study, we use a previously established approach that combines experiments and computational models to investigate the innate immune response during blood stream infections with the two fungal pathogens *C. albicans* and *C. glabrata*. First, we determine immune-reaction rates and migration parameters under healthy conditions. Based on these findings, we simulate virtual patients and investigate the impact of neutropenic conditions on the infection outcome with the respective pathogen. Furthermore, we perform *in silico* treatments of these virtual patients by simulating a medical treatment that enhances neutrophil activity in terms of phagocytosis and migration. We quantify the infection outcome by comparing the response to the two fungal pathogens relative to non-neutropenic individuals. The analysis reveals that these fungal infections in neutropenic patients can be successfully cleared by cytokine treatment of the remaining neutrophils; and that this treatment is more effective for *C. glabrata* than for *C. albicans*.

## Introduction

The human immune system protects the body against various environmental cues, such as microorganisms. It covers mechanisms on different levels ranging from physical barriers, like the skin and mucosal surfaces, down to cellular and molecular components of the innate and adaptive immune system (1). However, congenital or acquired diseases as well as medical treatments may impair proper functioning of the immune system, which can result in the loss of its protective ability. Neutrophils constitute the highest fraction of blood leukocytes, as they make up over 70% of all blood leukocytes (2). Since they can migrate to sites of infection and clear the organism from pathogens, they constitute an important part of the immune system.

*Candida* spp. cause 5–15% of all bloodstream infections and are associated with high mortality rates of 30–40% (3). A significant proportion (>50%, depending on the study setting) of the human population is colonized with *Candida* spp. The most prevalent species are *Candida albicans* and *Candida glabrata* that are both human commensals and reside predominantly on the human skin and mucosal surfaces (4–6). *C. albicans* is a morphotype-switching yeast, which in its commensal state exhibits the typical yeast form, while it forms hyphae when switching to its pathogenic state (7, 8). By contrast, *C. glabrata* does not form hyphae, neither in the commensal nor in the pathogenic state and is smaller than *C. albicans* (4, 9). In healthy people, both species usually stay in their commensal state. However, in immunocompromised patients, these human-pathogenic fungi can switch to their pathogenic state and cause superficial as well as systemic infections that are associated with high mortality rates.

To investigate host–pathogen interactions between the human innate immune system and these fungal pathogens, we applied a systems biology approach, where wet-lab experiments were combined with virtual infection models (10–13). Such virtual infection models have the great advantage of allowing for the identification and quantification of essential parameters that govern the biological system under consideration. This also makes them a powerful tool for hypothesis generation and uncovering new mechanisms, which consequently allows for minimizing the amount of animal experiments (14). Depending on the purpose, such *in silico* models can be built with different modeling techniques, such as *differential equations*, *state-based models* (SBMs) or spatial modeling techniques such as *cellular automata*, *cellular Potts models* or *agent-based models* (ABMs) (15). In a previous systems biology study, we established a human whole-blood infection assay (16), where blood was taken from healthy volunteers and infected with *C. albicans* cells. Then, subpopulations of alive, killed and extracellular fungal cells as well as fungal cells phagocytosed by monocytes and neutrophils were measured by association assays and survival assays. Based on these experimental data, we implemented an SBM that allowed for the quantification of immune-reaction rates, such as phagocytosis and killing rates, by fitting the simulated kinetics to the experimental data. In a subsequent study, we developed a bottom-up modeling approach that enabled not only quantification of immune-reaction rates but also the investigation of spatial aspects (17). Since the SBM simulates the temporal but not the spatial dynamics, we also developed an ABM that was based on a previous ABM implementation (18, 19). We combined both models in a bottom-up modeling approach (17): the SBM was used to determine non-spatial rates that were afterward transformed and used in the ABM to fit migration parameters of immune cells in human whole blood. We found that the *in silico* infection outcome for *C. albicans* was sensitive to changes in the diffusion coefficient of neutrophils, whereas that of monocytes had only minor impact on the system dynamics. This result reflected the more prominent role of neutrophils over monocytes in fighting *C. albicans* infection of human whole blood. Furthermore, immune dysregulation was investigated using the ABM, and the results showed that a reduced diffusion coefficient for neutrophils resembled conditions of neutropenia (17). This important observation is the main motivation of the present study, because it suggests how neutropenic patients may be treated to cope with bloodstream infections. Thus, increasing neutrophil activation in terms of phagocytic activity as well as migration strength is hypothesized to have the potential of balancing neutropenic conditions and clearance of infection. Based on this reasoning, we address infections in human whole blood by *C. albicans* and *C. glabrata* under neutropenic conditions in this study.

**Figure 1:**
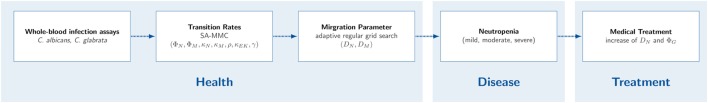
Workflow for studying neutropenia *in silico*. First, whole-blood infection assays with *Candida albicans* and *Candida glabrata* were performed in wet lab. Second, non-spatial immune-reaction rates were fitted using the state-based model. Third, the agent-based model (ABM) was used to estimate migration parameters for neutrophils and monocytes. Based on the fitted non-spatial immune-reaction rates and the fitted migration parameters, virtual neutropenic patients were simulated in the ABM by gradually reducing the neutrophil count. Eventually, a medical treatment of the virtual patients was simulated by increasing the diffusion coefficient and/or the phagocytosis rate of neutrophils.

Diseases or medical treatments can evoke a reduced absolute neutrophil count (ANC) in blood and result into a condition called *neutropenia*. Neutropenia may result from congenital or acquired impairments, where the latter case is more frequent. A reduced ANC may arise due to a disturbed development of neutrophils in the bone marrow, a disturbed migration to the blood stream or a rapid consumption during an infection (20). In anti-cancer chemotherapy, neutropenia is the most abundant disorder of the immune system due to the relatively short life-span of these terminally differentiated cells (21). Neutropenia emerges in different degrees of severity that are classified by the *Severe Chronic Neutropenia International Registry* (SCNIR) (20). The SCNIR distinguishes three degrees of severity: mild neutropenia with an ANC of 1,000–1,500 neutrophils/μl, moderate neutropenia with an ANC of 500–1,000 neutrophils/μl and severe neutropenia with an ANC of <500 neutrophils/μl. In this study, we focus on neutropenia treatment by stimulation and activation of present neutrophils by inflammatory cytokines and quantitatively investigate the impact on fungal infections by computer simulations. Thus, we aim to investigate a possible treatment strategy where the neutrophil activity is increased by a higher diffusion coefficient and/or phagocytosis rate. For this purpose, we apply the previously established protocol for whole-blood infection assays and perform the bottom-up modeling approach for the two human-pathogenic fungi. As is schematically shown in Figure 1, we first determine quantitative values for the immune-reaction rates as well as for diffusion coefficients of monocytes and neutrophils as the key immune cells of innate immunity in whole blood. Furthermore, we use this modeling approach to simulate neutropenia *in silico* and compare effects on the infection outcome between the different pathogens. To evaluate a possible treatment strategy, we simulate virtual neutropenic patients (VNP) with different degrees of severity and increase stepwise the phagocytosis rate and/or the diffusion coefficient of neutrophils to classify the infection outcome. Taken together, we could show that the increase of the phagocytosis rate and/or the migration parameter of neutrophils generally allowed balancing neutropenic conditions and clearance of infection. Furthermore, we predict that *C. albicans* compared with *C. glabrata* always requires stronger increases in the phagocytosis rate and the diffusion coefficient for the same conditions of neutropenia.

## Materials and Methods

### Ethics Statement

This study was conducted according to the principles expressed in the Declaration of Helsinki. All protocols were approved by the Ethics Committee of the University Hospital Jena (permit number: 273-12/09). Written informed consent was obtained from all blood donors.

### Fungal Strains and Culture

GFP expressing *C. albicans* strain [constructed as described in Ref. (16)] was grown in liquid yeast extract–peptone–dextrose (YPD) medium at 30°C. *C. glabrata* expressing GFP (22) was incubated at 37°C in YPD. In preparation for the whole-blood assay, both strains were reseeded after overnight culture in YPD medium and grown at 30 and 37°C, respectively, until they reached the mid-log-phase and finally harvested in HBSS until use.

### Whole-Blood Infection Assay

Human peripheral blood from healthy individuals was infected with either of the two fungi *C. albicans* and *C. glabrata*, respectively. The assay was performed as described previously (16). In short, 1 × 10^6^ *Candida* cells were added per ml of anti-coagulated blood and incubated at 37°C with gentle rotation for time points indicated. Following the incubation, cells were maintained at 4°C and analyzed immediately *via* flow cytometry. Flow cytometry gating strategy to investigate the distribution of fungal cells in human blood was performed as previously described (16) using FlowJo 7.6.4 software. Survival of fungal cells was determined in a plating assay by analysis of recovered colony-forming units after plating appropriate dilutions of all time points on YPD agar plates.

### Bottom-Up Modeling Approach

We established a bottom-up modeling approach for simulation and fitting of whole-blood infection assays in a previous study (17). This bottom-up modeling approach incorporates models with increasing complexity that build on one another, where each model focuses on different aspects of the infection process.

#### SBM—Immune-Reaction Rates

First, we applied the SBM to quantify and characterize immune-reaction rates for discrete entities of pathogens and innate immune cells. Therefore, the populations of innate immune cells, i.e., neutrophils and monocytes, as well as the pathogens were modeled by different states in the SBM. For the comparison with experimentally measured cell populations, we identified five combined units that are composed of specific states. The states representing extracellular cells are combined in the combined unit *P*_E_ that is given by the following equation:

(1) $$P_{\text{E}}\equiv P_{\text{AE}}+P_{\text{KE}}+P_{\text{AIE}}+P_{\text{KIE}},$$

where the states *P*_AE_ and *P*_KE_ represent extracellular cells that are alive and killed, respectively. The states *P*_AIE_ and *P*_KIE_ describe pathogens that are either alive and evading the immune response or killed and evading the immune response. Pathogens that are in extracellular space and either alive (*P*_AE_) or killed (*P*_KE_) can be phagocytosed by two different immune cells, i.e., neutrophils (N) and monocytes (M). The combined unit *P*_N_ comprises pathogens that are phagocytosed by neutrophils and is given by the following equation:

(2) $$P_{\text{N}}\equiv \sum_{i\geq 0}\sum_{j\geq 0}\left(i+j\right)N_{i,j}.$$

Similarly, pathogens that are phagocytosed by monocytes are combined in *P*_M_ that is given by the following equation:

(3) $$P_{\text{M}}\equiv \sum_{i\geq 0}\sum_{j\geq 0}\left(i+j\right)M_{i,j}.$$

In Eqs 2 and 3, the indices *i* and *j* refer to the immune cell state that is defined by the number of internalized alive and killed pathogens, respectively.

Furthermore, the states representing alive and killed pathogens are combined in *P*_K_ and *P*_A_, respectively, that are defined by the following equations:

(4) $$P_{\text{K}}\equiv P_{\text{KE}}+P_{\text{KIE}}+\sum_{i\geq 0}\sum_{j\geq 0}\left(M_{i,j}+N_{i,j}\right)\;j,$$

(5) $$P_{\text{A}}\equiv P_{\text{AE}}+P_{\text{AIE}}+\sum_{i\geq 0}\sum_{j\geq 0}\left(M_{i,j}+N_{i,j}\right)\;i.$$

The total number of pathogens is given by *P* ≡ *P*_E_ + *P*_N_ + *P*_M_ or *P* ≡ *P*_K_ + *P*_A_.

Transitions between these states are characterized by so-called *transition rates* and allow for dynamic state changes over time. The SBM of whole-blood infection comprises seven different transition rates that are given by the phagocytosis rate ϕ_M_ of monocytes, the phagocytosis rate ϕ_N_ of neutrophils, the intracellular killing rates κ_M_ and κ_N_ of both monocytes and neutrophils, the transition rates γ and ${\bar{\kappa }}_{\text{EK}}$, which define the extracellular killing by antimicrobial peptides, and the spontaneous immune evasion rate ρ. Note that, in the previous study by Lehnert et al. (17), a distinction between first and subsequent phagocytosis events by neutrophils was made, where the first phagocytosis event was assumed to activate the neutrophils and induce granulation. Since this fact is not experimentally validated for whole-blood infection with *C. glabrata*, we here did not distinguish between these two processes and used only one transition rate (ϕ_N_) referring to both first and subsequent phagocytosis events. To determine *a priori* unknown transitions rates, the *in silico* data were fitted to the experimental data by applying the method of *Simulated Annealing* based on the *Metropolis Monte Carlo* scheme (SA-MMC). For a more detailed description of the model and the parameter estimation method, we refer to Hünniger et al. (16) and Lehnert et al. (17).

#### ABM—Immune Cell Migration

The ABM is based on a previous ABM implementation (18, 19) and was already used in the previous study by Ref. (17). In contrast to the SBM, it allows studying spatial aspects, such as immune cell migration, in whole-blood infection assays. The ABM simulates all cell types, i.e., pathogens as well as immune cells, as individual spherical objects that are referred to as *agents*. All agents migrate, act and interact in a rule-based fashion within a spatially continuous, three-dimensional environment that represents 1 μl of blood.

Furthermore, the ABM was fitted to the experimental data to determine diffusion coefficients of neutrophils (*D*_N_) and monocytes (*D*_M_). This was done by the bottom-up modeling approach, where the previously determined transition rates from the SBM were used in the ABM. However, space-dependent rates, like phagocytosis rates, had to be adequately transformed (17). Regarding the fitting procedure, we used an *adaptive regular grid search* that scans the parameter space within reasonable ranges and uses a more fine-grained grid in regions with relatively small least squares errors (LSEs).

### Simulation Workflow

The work flow of this study, comparing wet-lab and *in silico* experiments with different models is displayed in Figure 1. First, we performed whole-blood infection assays for the two fungal pathogens *C. albicans* and *C. glabrata*. Afterward, we applied for each of the two pathogens the following steps. The results from association and survival assays were used to fit the model parameters of the SBM to these data. The transition rates of the fit with the lowest LSE were then appropriately transformed and fed into the ABM. Subsequently, the grid search in the parameter space was applied to fit the ABM to the experimental data and, in this way, to estimate the diffusion coefficients of neutrophils and monocytes. The determined transition rates and migration parameters form the basis for all following investigations on neutropenia and possible treatment strategies in virtual patients with varying degree of neutropenia. In the following, each step of this work flow is described in more detail.

#### Infection in Virtual Patients With Normal Neutrophil Counts

For the quantification of the immune response against the human-pathogenic fungi *C. albicans* and *C. glabrata* with normal neutrophil counts, we first determined the transition rates by fitting the SBM to the corresponding data from whole-blood experiments. These rates were used in the ABM and diffusion coefficients for neutrophils *D*_N_ and monocytes *D*_M_ were determined by fitting the ABM to the experimental data.

#### Infection in Virtual Patients Under Neutropenic Condition

To examine the immune response of virtual patients under conditions of neutropenia, we performed simulations with the immune-reaction rates and migration parameters that were identified under non-neutropenic conditions and gradually decreased the number of neutrophils. Subsequently, we compared the infection outcome at 4 h post infection for varying degrees of severity of neutropenia.

#### Patterns and Classification of Simulations

Since the health of a patient is critically determined by the amount of killed pathogens *P*_K_ as well as by the amount of alive and immune-evasive pathogens *P*_AIE_, we used these measures to characterize the infection outcome for the virtual patients.

We distinguish four different cases *C* for the infection outcome: an infection outcome corresponding to non-neutropenic immune conditions as well as the infection outcome under mild, moderate or severe neutropenia, i.e., *C *= {non–neutropenic, mild, moderate, severe}. To discriminate these classes, we calculated the patterns ψ = (μ(*P*_K_) ± σ(*P*_K_), μ(*P*_AE_) ± σ(*P*_AE_), μ(*P*_AIE_) ± σ(*P*_AIE_)) at the transition between consecutive degrees of neutropenia severity, in terms of the mean and SD. This resulted in the three patterns ψ = {ψ^nm^, ψ^mm^, ψ^ms^} at the transitions between two neutropenia severity levels: non-neutropenic–mild (nm), mild–moderate (mm), and moderate–severe (ms). For the classification of a particular simulation, we calculated the class of the values $P_{\text{K}}^{\text{sim}}$ and $P_{\text{AIE}}^{\text{sim}}$ at 4 h post infection. Then, we classified each of the three values of *v*(*P*_K_) = (μ(*P*_K_) + σ(*P*_K_), μ(*P*_K_), μ(*P*_K_) − σ(*P*_K_)) and *v*(*P*_AIE_) = (μ(*P*_AIE_) + σ(*P*_AIE_), μ(*P*_AIE_), μ(*P*_AIE_) − σ(*P*_AIE_)) separately. Thus, for each of the three values *v_i_*, we set:

(6) $$\begin{array}{llll} & C\left(v_{i}\left(P_{\text{K}}\right)\right)=\; & & \;\\ & \left\{\begin{array}{c} \;\mu \;\left(P_{\text{K}}^{\text{nm}}\right)-\sigma \;\left(P_{\text{K}}^{\text{nm}}\right)\leq v_{i}\leq 1,\;C=\text{non-neutropenic}\\ \;\mu \;\left(P_{\text{K}}^{\text{mm}}\right)-\sigma \;\left(P_{\text{K}}^{\text{mm}}\right)\leq v_{i}\leq \mu \;\left(P_{\text{K}}^{\text{nm}}\right)+\sigma \;\left(P_{\text{K}}^{\text{nm}}\right),\;C=\text{mild}\\ \mu \;\left(P_{\text{K}}^{\text{ms}}\right)\;-\;\sigma \;\left(P_{\text{K}}^{\text{ms}}\right)\leq v_{i}\leq \mu \;\left(P_{\text{K}}^{\text{mm}}\right)\;+\;\sigma \;\left(P_{\text{K}}^{\text{mm}}\right),\;C=\text{moderate}\\ \;0\;\leq v_{i}\leq \mu \;\left(P_{\text{K}}^{\text{ms}}\right)+\sigma \;\left(P_{\text{K}}^{\text{ms}}\right),\;C=\text{severe} \end{array}\right.\;,\; & \end{array}$$

(7) $$\begin{array}{llll} & C\left(v_{i}\left(P_{\text{AIE}}\right)\right)=\; & & \;\\ & \left\{\begin{array}{c} \;0\;\leq v_{i}<\mu \;\left(P_{\text{AIE}}^{\text{nm}}\right)+\sigma \;\left(P_{\text{AIE}}^{\text{nm}}\right),\;C=\text{non-neutropenic}\\ \mu \;\left(P_{\text{AIE}}^{\text{nm}}\right)-\sigma \;\left(P_{\text{AIE}}^{\text{nm}}\right)\leq v_{i}\leq \mu \;\left(P_{\text{AIE}}^{\text{mm}}\right)+\sigma \;\left(P_{\text{AIE}}^{\text{mm}}\right),\;C=\text{mild}\\ \mu \;\left(P_{\text{AIE}}^{\text{mm}}\right)\;-\;\sigma \;\left(P_{\text{AIE}}^{\text{mm}}\right)\;\leq \;v_{i}\;\leq \;\mu \;\left(P_{\text{AIE}}^{\text{ms}}\right)\;+\;\sigma \;\left(P_{\text{AIE}}^{\text{ms}}\right),\;C=\text{moderate}\\ \;\mu \;\left(P_{\text{AIE}}^{\text{ms}}\right)-\sigma \;\left(P_{\text{AIE}}^{\text{ms}}\right)\leq v_{i}<1,\;C=\text{severe} \end{array}\right.\;.\; & \end{array}$$

The simulation’s infection outcome *C* is then assigned to the class that received the highest number of votes from the nine values of *v_i_*(*P_K_*) and *v_i_*(*P*_AIE_).

#### *In Silico* Treatment of Neutropenia and Identification of Optimal Treatment Strategies

After the simulation of VNP, we simulated the medical treatment of these patients. Therefore, we selected virtual patients with certain degrees of severity of neutropenia. These are the number of neutrophils that are specific for a transition between two degrees of severity as well as the number of neutrophils between these transitions. Therefore, we simulate the following five VNP that are characterized by specific ANC: VNP-1 with 1,250 neutrophils/μl, VNP-2 with 1,000 neutrophils/μl, VNP-3 with 750 neutrophils/μl, VNP-4 with 500 neutrophils/μl, VNP-5 with 250 neutrophils/μl. Thus, the ANC of these VNP corresponds to a decrease in neutrophil number from the standard value by VNP-1: 75%, VNP-2: 80%, VNP-3: 85%, VNP-4: 90%, and VNP-5: 95%. Since the treatment with different drugs might improve the phagocytic activity and/or the migration parameter of neutrophils, we performed simulations with the ABM where the phagocytosis rate of neutrophils ϕ_N_ as well as their diffusion coefficient *D*_N_ was increased. In the following, we refer to these parameters that are affected by the treatment as $\phi_{\text{N}}^{\text{T}}$ and $D_{\text{N}}^{\text{T}}$.

For the sake of comparability of both values, we increased both values in a stepwise fashion. The increase of these values lead to an improvement in the infection outcome. For example, a virtual patient with moderate neutropenia and a simulated treatment might attain an infection outcome that corresponded to that of a patient with mild neutropenia or even to an infection outcome for an individual with a non-neutropenic immune status. Therefore, after simulating with a certain parameter set $\left(\phi_{\text{N}}^{\text{T}},\;D_{\text{N}}^{\text{T}}\right)$ we classified the simulation outcome as described earlier.

The stepwise increase of the parameters was continued until a parameter configuration was found with an infection outcome for non-neutropenic individuals. For quantification of the improvement of the infection outcome, we fitted an exponential function $f_{\phi_{\text{N}}}=1+a\cdot \;e^{-b\;\cdot f_{D_{\text{N}}}}$ at the transitions between two consecutive degrees of neutropenia severity. Here, the factors $f_{\phi_{N}}$ and $f_{D_{N}}$ are given by $f_{\phi_{\text{N}}}=\phi_{\text{N}}^{\text{T}}∕\phi_{\text{N}}^{\text{min}}$ and $f_{D_{\text{N}}}=D_{\text{N}}^{\text{T}}∕D_{\text{N}}^{\text{min}}$, and denote the ratios between the treatment parameter values $\left(\phi_{\text{N}}^{\text{T}},\;D_{\text{N}}^{\text{T}}\right)$ and the parameter values $\left(\phi_{\text{N}}^{\text{min}},\;D_{\text{N}}^{\text{min}}\right)$ obtained from minimizing the LSE under non-neutropenic conditions.

## Results

### Whole-Blood Infection Assays Differ for *C. albicans* and *C. glabrata*

**Figure 2:**
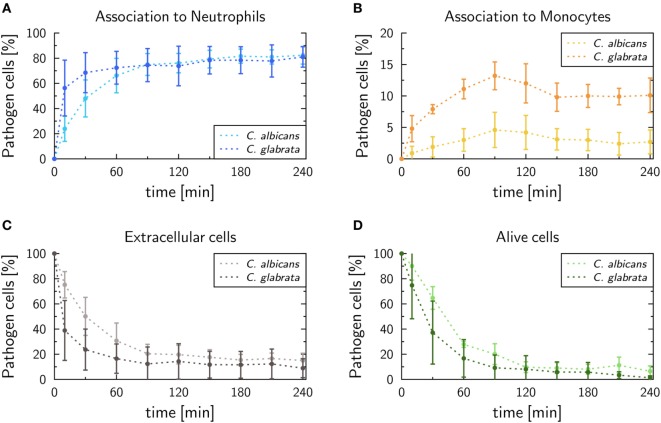
Experimental data of whole-blood infection assays for *Candida albicans* (light color) and *Candida glabrata* (dark color), respectively. After incubation populations of extracellular cells **(A)**, alive cells **(B)**, as well as pathogens phagocytosed by either neutrophils **(C)** or monocytes **(D)**, were measured by flow cytometry and plating assays.

In this study, we performed human whole-blood infection assays with *C. glabrata* and compared the measured data with experimental measurements for *C. albicans* by applying a previously established protocol (16). The kinetics of pathogens associated with either neutrophils or monocytes can be seen in Figures 2A,B, respectively. In case of *C. glabrata*, 81.0 ± 8.1% cells were associated with neutrophils, which is similar to *C. albicans* with 82.3 ± 7%. However, the experimental data show different kinetics for the two species, since *C. glabrata* is phagocytosed by neutrophils in a shorter time. By contrast, the association with monocytes is higher for *C. glabrata* with 10.1 ± 2.7%, while only 2.7 ± 1.9% *C. albicans* cells were associated with monocytes 4 h post infection. Due to the phagocytosis of the pathogens by the immune cells, 4 h post infection, 8.9 ± 7.5% cells remained extracellular for *C. glabrata* and 15.0 ± 5.8% for *C. albicans* (see Figure 2C). The remaining extracellular cells are referred to as *immune-evasive* cells, as already introduced in previous studies (16, 17). Furthermore, 1.3 ± 1.5% *C. glabrata* cells remained extracellular and alive 4 h post infection (see Figure 2D), which is lower compared with *C. albicans* with 6.5 ± 4.2%. In comparison with *C. albicans*, the decrease in alive *C. glabrata* cells mainly occurred during the first 2 h of the experiment exhibiting a much faster kinetics than for *C. albicans*.

### Quantification of Immune-Reaction Rates Reveals Differences Between Pathogens

**Figure 3:**
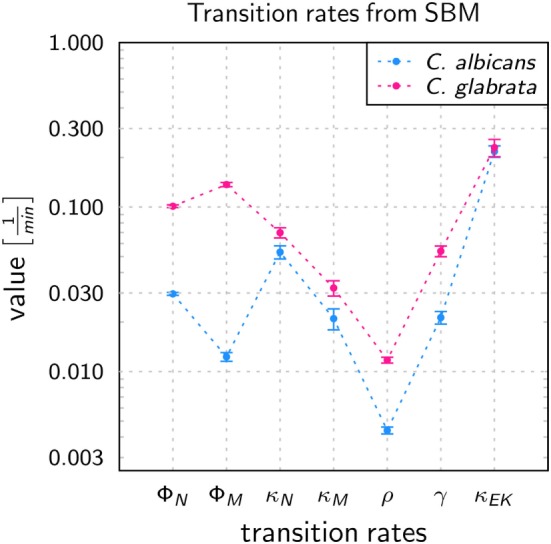
Transition rates obtained from the calibration of the state-based model (SBM) to experimental data of the whole-blood infection assay for *Candida albicans* (blue) and *Candida glabrata* (pink), respectively. The values are compared for the phagocytosis rate for neutrophils (ϕ_N_), and by monocytes (ϕ_M_), killing rate for neutrophils (κ_N_) and monocytes (κ_M_), the rate at which the pathogens can evade the immune response with regard to phagocytosis and/or killing (ρ) as well as the rates that define the extracellular killing, i.e., γ and ${\bar{\kappa }}_{\text{EK}}$. Error bars correspond to SDs.

To quantify infection scenarios for the two pathogens, immune-reaction rates of the SBM were estimated by fitting to the experimental data as done previously for *C. albicans* in human whole blood (17). As explained in detail in Section “Material and Methods,” this was done by computing the so-called *combined units*, which are combinations of different pathogen states and were directly accessible in experiment. In terms of these combined units, we evaluated the quality of a simulation by calculating the LSE between the experimental data and the *in silico* data. To determine the immune-reaction rates representing the best fit to the experimental data, i.e., that are associated with the lowest LSE, we applied the method of *Simulated Annealing* based on the *Metropolis Monte Carlo* scheme. The resulting immune-reaction rates from the fitting procedure where used to simulate the infection with the pathogens in 1 ml of blood, containing 5 × 10^6^ neutrophils, 5 × 10^5^ monocytes, and 1 × 10^6^ cells, and are shown in Figure 3 and in Table S1 in Supplementary Material.

The values of immune-reaction rates for *C. albicans* infection of whole blood are in line with our previous results (17). The reaction rate values for *C. glabrata* infection mostly differ in comparison to reaction rates for *C. albicans* infection (see Figure 3). The phagocytosis rate of neutrophils in the infection scenario with *C. glabrata* is ϕ_N_ = 10.11 × 10^−2^ min^−1^, which is 3.5 times higher than for *C. albicans* infection. The phagocytosis rate for monocytes is with ϕ_M_ = 13.69 × 10^−2^ min^−1^ an order of magnitude higher than in the case of *C. albicans* infection. These higher phagocytosis rates arise due to the faster kinetics measured for *C. glabrata* in the experimental data (see Figure 2). Furthermore, the order in the magnitude of phagocytosis rates is reversed in comparison to *C. albicans* infection, because for *C. glabrata* the phagocytosis rate of monocytes is 1.4 times higher than that for neutrophils. The killing rate of neutrophils is for *C. glabrata* κ_N_ = 6.98 × 10^−2^ min^−1^, which is only slightly higher than for *C. albicans* infection. Furthermore, differences between the fungal pathogens are again observed in the killing rate for monocytes, which is 1.5 times higher for *C. glabrata* with κ_M_ = 3.22 × 10^−2^ min^−1^ compared with *C. albicans*. As was previously observed for *C. albicans* (16, 17), also *C. glabrata* was found to evade the immune response and to remain even hours post infection alive and non-phagocytosed in human whole blood (Figures 2C,D). The rate for fungal cells becoming evasive against the immune response is for both pathogens comparably low, i.e., ρ = 1.173 × 10^−2^ min^−1^ for *C. glabrata* and ρ = 0.439 × 10^−2^ min^−1^ for *C. albicans*. A comparison of both rates that define the extracellular killing by antimicrobial peptides (κ_EK_(*t*)) showed that the value of ${\bar{\kappa }}_{\text{EK}}$ is similar for both pathogens (see Table S1 in Supplementary Material) and γ is 2.5 times larger for infection scenarios with *C. glabrata* (γ = 5.39 × 10^−2^ min^−1^).

The time-resolved kinetics of the fits with the lowest LSE for the two fungal pathogens can be seen in Figures S1 and S2 in Supplementary Material, where the thickness of the simulation curves reflect random variations within the SDs of the immune-reaction rates. For both pathogens, the SBM adequately resembled the experimental data. Since the SBM neglects all spatial aspects of the infection scenarios, we performed a bottom-up modeling approach by combining the SBM with the ABM (17).

### Migration Parameters of Phagocytes in Response to Various Pathogens Differ Quantitatively

**Figure 4:**
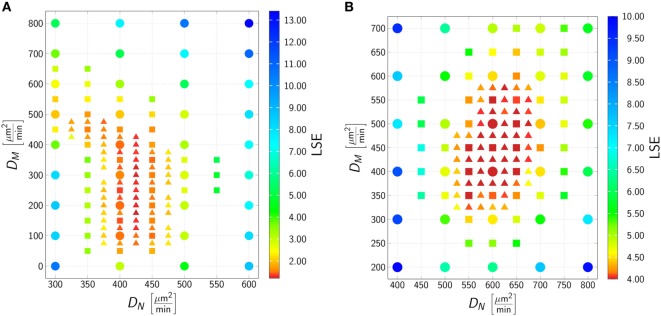
Result of the agent-based model (ABM) parameter estimation for whole-blood infection assays with *Candida albicans* **(A)** and *Candida glabrata* **(B)**. Adaptive regular grid search was applied to fit the ABM to the experimental data and diffusion coefficients for neutrophils (*D*_N_) and monocytes (*D*_M_) were determined. At each grid point 1 μl blood was simulated, and 30 realizations for each parameter configuration were performed. Three different refinement levels were performed: simulations of the first level are represented as dots, simulations of the second level are represented as squares, and simulations of the third level are represented as triangles. The best fit to the experimental data was found at $\left(D_{\text{N}}^{\text{min}},\;D_{\text{M}}^{\text{min}}\right)=\left(\text{425 }\mu {\text{m}}^{\text{2}}∕\text{min},\text{175}\;\mu {\text{m}}^{\text{2}}∕\text{min}\right)$ for *C. albicans* and at $\left(D_{\text{N}}^{\text{min}},\;D_{\text{M}}^{\text{min}}\right)=(\text{600}\;\mu {\text{m}}^{\text{2}}∕\text{min},$ $\text{425}\;\mu {\text{m}}^{\text{2}}∕\text{min})$ for *C. glabrata*.

To determine the migration parameters of neutrophils and monocytes in whole-blood infection scenarios with the respective pathogens, we used the experimentally measured data as well as the fitted immune-reaction rates from the SBM to perform stochastic spatiotemporal simulations by the ABM in 1 μl of blood. As a result of this bottom-up modeling approach for whole-blood infection assays, we obtained the diffusion coefficients of the immune cells in response to *C. albicans*. This can be seen in Figure 4A, where the best solution, i.e., the parameter configuration of (*D*_N_, *D*_M_) that resulted in the smallest LSE, was identified to be $\left(D_{\text{N}}^{\text{min}},\;D_{\text{M}}^{\text{min}}\right)=\left(425\;\mu {\text{m}}^{2}∕\text{min},175\;\mu {\text{m}}^{2}∕\text{min}\right)$. In line with our earlier findings (17), for *C. albicans* the LSE was sensitive for variations in *D*_N_ but not for variations in *D*_M_. The range of *D*_M_ that still lead to comparably low LSE values spans from approximately 100 μm^2^/min up to 500 μm^2^/min, whereas the range with comparably low LSE for *D*_N_ was limited to 400–425 μm^2^/min. As shown in Figure S3 in Supplementary Material, the fitting results are in excellent agreement with the experimental data, and the stochasticity of the *in silico* experiments still give rise to low SDs in the simulation curves, as can be inferred from the thickness of the curves representing 30 runs.

The best fit of the simulation curves to the experimental data of whole-blood infection assays for *C. glabrata* was achieved for diffusion coefficients for neutrophils and monocytes with values $\left(D_{\text{N}}^{\text{min}},\;D_{\text{M}}^{\text{min}}\right)=\left(600\;\mu {\text{m}}^{2}∕\text{min},425\;\mu {\text{m}}^{2}∕\text{min}\right)$ (see Figure 4). We note that the range in which the diffusion coefficient of monocytes can vary for comparable LSE values was found to be much more restricted than in the case of *C. albicans*, i.e., this range for *D*_M_ was from 350 μm^2^/min up to 575 μm^2^/min for fitting results with comparable LSE. However, in the case of *C. glabrata*, neutrophils were not found to be restricted to the small range of only ± 12 μm^2^/min as for *C. albicans*, but could vary in a range of ± 80 μm^2^/min. As can be seen in Figure S4 in Supplementary Material, the experimentally determined kinetics of the infection scenario with *C. glabrata* is in excellent agreement with the simulation curves of the ABM.

### Immune Response in Virtual Patients With Neutropenia Is Strongly Pathogen Dependent

Our previous considerations reveal that immune cells exhibit a qualitatively and quantitatively different response against *C. albicans* and *C. glabrata* in human whole-blood infection assays. Comparing *C. glabrata* to *C. albicans* infection, this is reflected by (i) increased phagocytosis rates and (ii) increased diffusion coefficients by factors of 1.4 and 2.4, respectively, for neutrophils and monocytes. In line with our previous work on the comparison between *C. glabrata* with *C. albicans* by live-cell imaging of phagocytosis assays (23–26), these quantitative differences are accompanied with the qualitative variation in the immune response that involves much stronger monocyte activation in the case of *C. glabrata*. Nevertheless, a prominent role is played by neutrophils that are quantitatively prevalent in cell number and qualitatively important in differently directing the immune response against these fungal pathogens (23).

**Figure 5:**
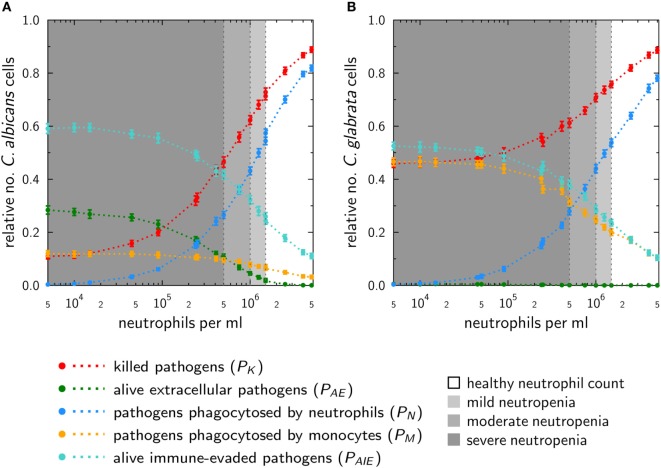
*In silico* infections under neutropenic conditions with *Candida albicans* **(A)** and *Candida glabrata* **(B)** were performed by gradually decreasing the absolute neutrophil count in the agent-based model. Plots show the fraction of killed cells (red), alive and extracellular cells (green), phagocytosed cells by neutrophils (blue), and monocytes (yellow) as well as (alive) cells that are able to evade the immune system (turquoise) at 4 h post infection.

To investigate the impact of neutropenia on the infection outcome with a specific pathogen, we simulated VNP using the ABM. Here, the optimal immune-reaction rates and diffusion coefficients were used as previously determined for normal ANC values. In the virtual patients, we stepwise decreased the number of neutrophils to resemble different degrees of severity of neutropenia and simulated the early immune response during 4 h post infection. The contributions of the combined units—such as killed, phagocytosed and immune-evasive *Candida* cells at 4 h post infection—are shown in Figure 5.

The phagocytosis by neutrophils is for both pathogens quite similar. For mild neutropenia the phagocytosis by neutrophils ranges for both fungal pathogens between ~40 and 50%, for mild neutropenia between ~25 and 40%, and is below ~25% for severe neutropenia. Interestingly, despite these similarities, the infection outcomes for the two pathogens under the condition of neutropenia are predicted to be remarkably different. As shown in Figure 5A, a stronger impact on the infection outcome can be observed for *C. albicans*, where in the scenario of severe neutropenia the number of killed fungal cells achieves only 10–45%. By contrast, killing of *C. glabrata* in severe neutropenia is more efficient, and the fraction of dead cells ranges between 45 and 60% of total fungal cells (see Figure 5B).

This difference is governed by the behavior of monocytes in response to the two fungal pathogens. Higher phagocytosis rates in case of *C. glabrata* compared with *C. albicans* enable monocytes to partially compensate for the loss of neutrophils under conditions of neutropenia. This compensatory effect is relatively low for *C. albicans*, where the fraction of cells that were phagocytosed by monocytes increased from 3% for normal ANC to only 12% under the condition of severe neutropenia (see Figure 5A). For *C. glabrata*, this increase in monocyte phagocytosis rose from 10 to 46% of the *C. glabrata* cells (see Figure 5B). Furthermore, the infection outcome is also characterized by the number of cells that are able to evade the immune response. Immune evasion is more pronounced for *C. albicans*, where also for normal ANC 15% of all fungal cells are able to evade the immune response (see Figure 5A). However, with stronger degrees of neutropenia the fraction of these cells even increases to about 60%. In the case of *C. glabrata*, only 10% of the cells can evade the immune response for normal ANC, while this fraction rises up to 50% under conditions of severe neutropenia (see Figure 5B). As explained in Section “Materials and Methods,” the infection outcome is mainly characterized by the fraction of killed as well as the fraction of alive and immune-evasive *Candida* cells. Therefore, we assigned the values of *P*_K_ and *P*_AIE_ at the boundaries to pattern that characterize the different degrees of severity of neutropenia (see Table S2 in Supplementary Material). Subsequently, with the help of these patterns, we were able to classify simulations of medical treatments in neutropenic patients.

### Simulation of Medical Treatment for VNP

After we simulated the infection with the pathogens *C. albicans* and *C. glabrata* in VNP, we selected five types of VNP with different severity degrees of neutropenia for *in silico* treatment. The VNP-1 is characterized by an ANC of 1,250 neutrophils/μl representing patients with mild neutropenia. At the transition between mild and moderate, the ANC is 1,000 neutrophils/μl, and the corresponding VNP is referred to as VNP-2. Similarly, we defined VNP-3, VNP-4 and VNP-5 that are characterized, respectively, by ANC of 750 neutrophils/μl (moderate neutropenia), 500 neutrophils/μl (transition between moderate and severe neutropenia), and 250 neutrophils/μl (severe neutropenia). The *in silico* treatment involves the increase of neutrophil activation in terms of their phagocytosis rate and/or diffusion coefficient to quantitatively investigate its impact on the reduced numbers of neutrophils in these patients. Thus, increasing the phagocytosis rate and/or diffusion coefficient of neutrophils in a stepwise fashion, we simulated the infection with either of the two pathogens *C. albicans* and *C. glabrata* under neutropenic conditions. Afterward, the infection outcome of the simulation was classified according to the previously determined pattern (see Patterns and Classification of Simulations). To find a formal description of the increase of neutrophil phagocytosis rate and diffusion coefficient required for reaching the infection outcome for non-neutropenic individuals, we fitted an exponential function of the form $f_{\phi_{\text{N}}}=1+a\cdot e^{-b\;\cdot f_{D_{\text{N}}}}$ at the transition where the non-neutropenic infection outcome is reached. Here, the factors $f_{\phi_{N}}$ and $f_{D_{N}}$ are defined as $f_{\phi_{\text{N}}}=\phi_{\text{N}}^{\text{T}}∕\phi_{\text{N}}^{\text{min}}$ and $f_{D_{\text{N}}}=D_{\text{N}}^{\text{T}}∕D_{\text{N}}^{\text{min}}$, where $\phi_{\text{N}}^{\text{T}}$ and $D_{\text{N}}^{\text{T}}$ denote parameters that are affected by the treatment, and $\phi_{\text{N}}^{\text{min}}$ and $D_{\text{N}}^{\text{min}}$ refer to the parameter values obtained by minimizing the LSE under non-neutropenic conditions. We varied $\phi_{\text{N}}^{\text{T}}$ and $\;D_{\text{N}}^{\text{T}}$ over one order of magnitude, i.e., $f_{\phi_{\text{N}}},f_{D_{\text{N}}}\in \left[1,10\right]$, and plotted the resulting curves for each type of VNP in Figure S5 in Supplementary Material for the fitting parameters *a* and *b* as provided in Table S3 in Supplementary Material.

**Figure 6:**
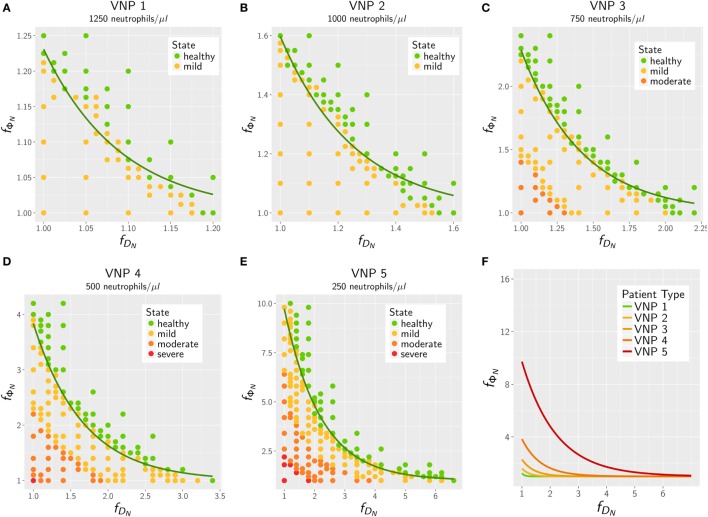
*In silico* treatment of virtual neutropenic patients (VNP) infected with *Candida albicans* was simulated using the agent-based model. Stepwise increase of phagocytosis rate and diffusion coefficient of neutrophils was performed for VNP with various severity degrees of neutropenia: VNP1–5: 1,250 **(A)**, 1,000 **(B)**, 750 **(C)**, 500 **(D)**, and 250 **(E)** neutrophils/μl. Simulated points are classified according to the previously determined patterns: green points show a non-neutropenic infection outcome, yellow points show an infection outcome comparable to a mild neutropenia, orange points show an infection outcome comparable to a moderate neutropenia, and red points show an infection outcome comparable to a severe neutropenia. Solid lines depict the fitted exponential function $f_{\Phi_{\text{N}}}=\text{1}+a\cdot e^{-b\cdot f_{D_{\text{N}}}}$ at the transition to the non-neutropenic infection outcome. For comparison the fitted curves for the five VNP with their severity degrees of neutropenia are shown in panel **(F)**.

**Figure 7:**
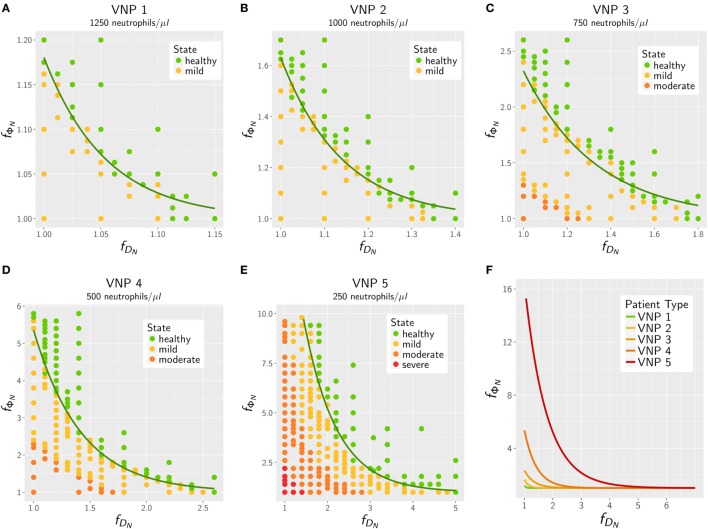
*In silico* treatment of virtual neutropenic patients (VNP) infected with *Candida glabrata* was simulated using the agent-based model. Stepwise increase of phagocytosis rate and diffusion coefficient of neutrophils was performed for VNP with various severity degrees of neutropenia: VNP1–5: 1,250 **(A)**, 1,000 **(B)**, 750 **(C)**, 500 **(D)**, and 250 **(E)** neutrophils/μl. Simulated points are classified according to the previously determined patterns: green points show a non-neutropenic infection outcome, yellow points show an infection outcome comparable to a mild neutropenia, orange points show an infection outcome comparable to a moderate neutropenia, and red points show an infection outcome comparable to a severe neutropenia. Solid lines depict the fitted exponential function $f_{\Phi_{\text{N}}}=\text{1}+a\cdot e^{-b\cdot f_{D_{\text{N}}}}$ at the transition to the non-neutropenic infection outcome. For comparison, the fitted curves for the five VNP with their severity degrees of neutropenia are shown in panel **(F)**.

**Figure 8:**
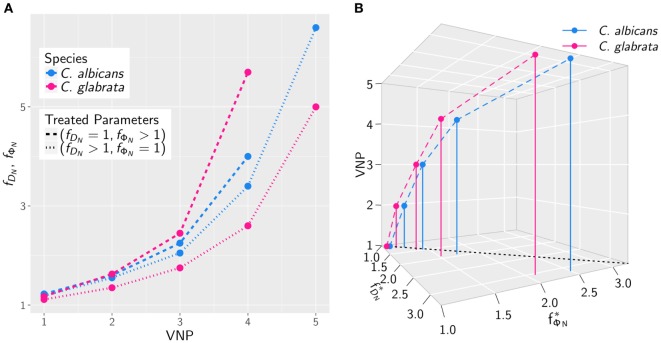
The increase in neutrophil activation required to reach the infection outcome of non-neutropenic patients depends on the severity degree of neutropenia in VNP. **(A)** Comparison of *Candida albicans* (blue) and *Candida glabrata* (pink) infection for various VNP in terms of the factors $f_{D_{\text{N}}}$ and $f_{\phi_{\text{N}}}$ keeping either $f_{\phi_{\text{N}}}=\text{1}$ or $f_{D_{\text{N}}}=\text{1}$ fixed. **(B)** The same as in panel **(A)** allowing both factors to vary to attain the optimal values $\left(f_{\phi_{\text{N}}}^{*},f_{D_{\text{N}}}^{*}\right)$ with minimal distance from $\left(f_{\phi_{\text{N}}\;}=\text{1},f_{D_{\text{N}}\;}=\text{1}\right)$ at which the infection outcome of non-neutropenic patients is reached.

The results for the *in silico* treatment of VNP with *C. albicans* and *C. glabrata* infection are shown in detail in Figures 6 and 7, respectively. Performing more than 4 × 10^4^ simulations, we generally found that all VNP do reach the infection outcome of non-neutropenic patients by increasing neutrophil activation in terms of phagocytosis rate and/or diffusion coefficient. As could be expected, the required increase in neutrophil activation depends on the severity degree of neutropenia in VNP. For VNP with severe neutropenia (VNP-5), reaching the infection outcome of non-neutropenic patients would require relatively high values for $\phi_{\text{N}}^{\text{T}}$ with $f_{\phi_{N}}>10$, whereas the treatment was always successful for $D_{\text{N}}^{\text{T}}$ with $f_{D_{\text{N}}}\ll 10$. To compare the two fungal pathogens with each other, we first fixed either $\phi_{\text{N}}^{\text{T}}=\phi_{\text{N}}^{\text{min}}$ $\left(f_{\phi_{N}}=1\right)$ or $D_{\text{N}}^{\text{T}}=D_{\text{N}}^{\text{min}}$ $\left(f_{D_{\text{N}}}=1\right)$ and varied only one parameter, respectively, $D_{\text{N}}^{\text{T}}$ or $\phi_{\text{N}}^{\text{T}}$. As can be seen in Figure 8A, for both fungal pathogens increasing the diffusion coefficient yields the infection outcome of non-neutropenic patients at smaller factors than increasing the phagocytosis rate, i.e., $f_{D_{\text{N}}}<f_{\phi_{\text{N}}}$. Interestingly, increasing only the neutrophil diffusion, the *in silico* treatment was found to be more effective for *C. glabrata*, whereas it turned out to be more effective for *C. albicans* if only the phagocytosis rate was increased. The combined impact of increasing $\phi_{\text{N}}^{\text{T}}$ and $D_{\text{N}}^{\text{T}}$ yielded a pair $\left(f_{\phi_{\text{N}}}^{\;*},f_{D_{\text{N}}}^{\;*}\right)$ of optimal values with minimal distance from $\left(f_{\phi_{\text{N}}}=1,f_{D_{\text{N}}}=1\right)$ where the infection outcome of non-neutropenic patients was reached. The results are shown in Figure 8B, where the comparison between *C. albicans* and *C. glabrata* predicts that $f_{\phi_{\text{N}}}^{\;*}<f_{D_{\text{N}}}^{\;*}$ for the optimal *in silico* treatment, i.e., the required relative increase of the diffusion coefficient is larger than that for the phagocytosis rate. Moreover, the optimal *in silico* treatment was reached for factors $\left(f_{\phi_{\text{N}}}^{\;*},f_{D_{\text{N}}}^{\;*}\right)$ with lower values for all VNP in the case of *C. glabrata*.

## Discussion

In this study, we investigated bloodstream infections with the fungal pathogens *C. albicans* and *C. glabrata* in human whole blood. Special focus was put on the infection scenario under neutropenic conditions as well as possible treatment strategies. These conditions are clinically relevant as it is well established that neutropenia promotes dissemination of *Candida* spp. during bloodstream infection and impairs prognosis. We used a previously established bottom-up modeling approach that combines different mathematical modeling approaches of increasing complexity based on wet-lab experiments (17). To investigate infection by different fungal pathogens, we first performed whole-blood infection assays using blood of healthy individuals. In the past, these whole-blood infection models have already been successfully applied to analyze the early immune response to clinically relevant pathogens (27–29) and to identify their virulence factors (30, 31). Furthermore, the influence of genetic polymorphisms on the immune response have been tested (32, 33) as well as potential therapeutic approaches and vaccine efficacy (34–38). In this study, we applied this experimental modeling approach to investigate early immune responses to the two *Candida* spp. in blood. The resulting experimental data showed that the immune response followed a faster kinetics for *C. glabrata* than for *C. albicans*, which is reflected by an earlier phagocytosis of this pathogen. In line with our previous studies (16, 17, 23), monocytes were found to contribute more to the immune response against *C. glabrata* compared with *C. albicans*.

The system behavior was quantified by estimating values for immune-reaction rates, such as phagocytosis and killing rates, based on fitting a SBM to the experimentally measured data (17). As expected from the observed difference in the kinetics of the immune response between *C. albicans* and *C. glabrata*, we found that the phagocytosis rates were orders of magnitude higher for *C. glabrata* with monocytes reaching the highest values (see Table S2 in Supplementary Material). Thus, for *C. glabrata* the phagocytosis rate for monocytes is higher than for neutrophils and this relation is inverted for *C. albicans*. Applying a bottom-up modeling approach (17), we used an ABM to estimate migration parameters for neutrophils and monocytes in response to the two fungal species. For *C. glabrata* these migration parameter were higher than for *C. albicans*. As previously shown for *C. albicans* the outcome of the immune response was restricted to a narrow regime of migration parameters for the neutrophils (17), whereas these migration parameters in the case of *C. glabrata* infections could vary over a significantly wider range to fit the experimental data. This is another indication for the observable fact that monocytes play a more important role in the defense against *C. glabrata* compared with *C. albicans* (23, 39).

Since fungal infections by *Candida* spp. are a major risk for immunocompromised patients, we extended the computer simulations for normal ANC by numerically studying infection scenarios in virtual patients with different severity degrees of neutropenia. Due to the pronounced importance of neutrophils in the immune response against *C. albicans*, these computer simulations predicted a strong negative impact on the infection outcome for VNP depending on the severity degree of neutropenia. Although the impact of neutropenia on the infection outcome during *C. glabrata* infection was not as strong as for *C. albicans*, the immune response was still to a large extent impaired. For example, this was observed by the prediction that the fraction of killed pathogens at 4 h post infection decreased from around 90% for both species under normal ANC to about 50 and 10% for *C. glabrata* and *C. albicans* for severe neutropenic conditions, respectively. Moreover, at 4 h post infection, a fraction of 30% *C. albicans* cells are still alive and extracellular in human blood that could contribute to the dissemination to other body parts in real patients. While the fraction of alive and extracellular *C. glabrata* cells is negligible at 4 h post infection, a large fraction of about 50% is phagocytosed by monocytes including a few percent of fungal cells that are still alive and may disseminate by eventually escaping from the monocytes. These data again point toward different virulence traits in the two *Candida* spp. (40).

The bottom-up modeling approach for the simulation of infection scenarios under neutropenic conditions was established to simulate the effects of medical treatments. To date there exist three different ways to approach neutropenia in the clinical setting, which comprise (i) the stimulation and activation of remaining neutrophils by medical treatment of the patient, (ii) the internal stimulation of neutrophil maturation and release from the bone marrow by medication of patients with *granulocyte colony-stimulating factor* (G-CSF), and (iii) the transfusion of G-CSF/steroid mobilized neutrophils from a donor. The latter treatment of healthy donors leads to a vast increase of peripheral blood neutrophils (41–44), which are subsequently extracted from the donor by leukapheresis and administered to the patient to increase the ANC in blood. This therapy shows higher rates of patient survival in the context of bacterial infections (43), whereas improvement in patient survival was not consistently observed for fungal infections (45–47). In particular, Gazendam et al. (48) show that the G-CSF/dexamethasone stimulation of donor neutrophils leads to a change in their granular content, which impairs the fungal killing capacity with regard to *C. albicans*. The cytokine treatment with G-CSF to trigger the neutrophil release from the bone marrow in patients is mainly applied in congenital neutropenia and causes a significant increase of the ANC in blood (49, 50). Before effective drugs were available, children with congenital neutropenia typically died in their first year of life due to bacterial and fungal infections (51, 52). The G-CSF treatment makes use of the emergency mobilization of neutrophils in response to an inflammatory signal and the secretion of chemokines leading to neutrophil migration into blood vessels (53). However, patients can be also *low-responders* or even *non-responders* exhibiting reduced effects of G-CSF (49, 54). Finally, instead of increasing the circulating number of neutrophils, the option to medically treat neutropenia by inflammatory cytokines, such as *interferon* γ and *tumor necrosis factor* α, yields a modulation of the immune response by the stimulation and activation of neutrophils in blood (41, 44). Both cytokines have been reported to enhance the neutrophil response against fungi, e.g., *Candida* spp. (55), *Aspergillus* spp. (56), and *Cryptococcus* spp. (57).

In this study, we focused on investigating the treatment of neutropenic patients by inflammatory cytokines to quantify the possibility of balancing neutropenic conditions and clearance of infection. The simulations of this *in silico* treatment revealed that an increase of the phagocytosis rate and/or the migration parameter of neutrophils generally improved the infection outcome. For both *Candida* spp. under investigation, conditions of mild neutropenia can be compensated resembling an infection outcome of non-neutropenic individuals by an increase in either the phagocytosis rate or the diffusion coefficient, or a combination of both, by less than 25% percent. The computer simulations allowed us to rigorously quantify the relative change in these parameters needed for any severity level of neutropenia. In the case of severe neutropenia, medical treatments would need to increase these parameters by at least 250% for the phagocytosis rate and at least 300% for the diffusion coefficient to reach infection outcomes in VNP comparable to individuals with normal ANC. It should be noted that the modulation of parameters has to be combined, because even a 10-fold increase of the phagocytosis rate alone would not recover the infection outcome of non-neutropenic individuals. Thus, the quantitative simulation of *in silico* treatments generates concrete predictions regarding the relative impact that treatments with inflammatory cytokines are required to exert on these two parameters. Moreover, our numerical experiments predict that *C. albicans* compared with *C. glabrata* always requires stronger increases in the phagocytosis rate and the diffusion coefficient for the same conditions of neutropenia.

Clearly, the underlying model assumptions (such as spatial homogeneity and absence of external forces) cannot be 1:1 translated into the *in vivo* situation—neither in small vessels nor in tissue. Despite this, several predictions resulting from the model could be confirmed *in vivo* or are in line with clinical findings (16). For this study, this also applies to the observations that (i) neutropenia may result in poor prognosis and a higher ratio of disseminated candidiasis [e.g., Ref. (58, 59)] and (ii) monocytes play a more important role in *C. glabrata* infection (23). Even though clinical studies will ultimately be required to validate our hypotheses, the first step would be to test these treatment strategies in whole-blood infection assays and our simulations for VNP can be used for this testing.

Our study may be extended in different ways. For example, computer simulations for various pathogens, such as *Staphylococcus* spp. and *Streptococcus* spp., which were shown to cause bacteremia and sepsis under conditions of neutropenia, could be performed (52, 60). Moreover, treatment strategies that lead to an increased ANC in neutropenic patients, like the transfusion therapy as well as the G-CSF treatment, could be simulated and compared with the cytokine treatment considered in this study. Furthermore, the bottom-up approach provides the possibility to investigate the impact of other immune disorders on the infection outcome with the pathogens under consideration. Moreover, the generated predictions of this study could be examined in future wet-lab experiments. Therefore, whole-blood infection assays with *C. albicans* or *C. glabrata* in human blood with reduced ANC could be performed. Such neutropenic blood samples could be taken from patients with neutropenia, where it should be considered that primary diseases of the patient may affect the experimental results. Another possibility may be to generate neutropenic blood samples in the wet lab by a controlled reduction of the neutrophil number. However, this poses a high challenge, since the remaining blood constituents will be affected by side effects that cannot be well controlled. Investigating such host–pathogen interactions by combining wet-lab and dry-lab studies is in the spirit of system biology. This approach provides a powerful tool to investigate biological systems in a qualitative as well as quantitative fashion and enables hypothesis generation in dry-lab as well as hypothesis testing in wet-lab studies.

## Ethics Statement

Human peripheral blood was collected from healthy volunteers after informed consent. This study was conducted according to the principles expressed in the Declaration of Helsinki. All protocols were approved by the Ethics Committee of the University Hospital Jena (permit number: 273-12/09).

## Author Contributions

ST and MTF conceived and designed this study. MTF and OK provided computational resources and materials, respectively. Data processing, implementation, and application of the computational algorithm were done by ST, TL, MP, and MTF. Experiments were performed by KH and IL. ST, TL, MP, KH, IL, OK, and MTF evaluated and analyzed the results of this study; drafted the manuscript and revised it critically for important intellectual content and final approval of the version to be published; and agreed to be accountable for all aspects of the work in ensuring that questions related to the accuracy or integrity of any part of the work are appropriately investigated and resolved.

## Conflict of Interest Statement

The authors declare that the research was conducted in the absence of any commercial or financial relationships that could be construed as a potential conflict of interest.
