# Supplementary material for: Quantitative Simulations Predict Treatment Strategies Against Fungal Infections in Virtual Neutropenic Patients
Source: Front Immunol. 2018 Apr 4;9:667. doi: 10.3389/fimmu.2018.00667 (PMC5893870; doi:10.3389/fimmu.2018.00667)
Supplement: Supplementary file 1 [file data_sheet_1.docx]

Supplementary Material

Quantitative simulations predict treatment strategies against fungal infections in virtual neutropenic patients

**Sandra Timme^1,2^, Teresa Lehnert^1,3^, Maria T. E. Prauße^1,2^, Kerstin Hünniger^4,5^, Ines**

**Leonhardt^3,4^, Oliver Kurzai^3,4,5^, and Marc Thilo Figge^1,2,3,*^**

^1^Research Group Applied Systems Biology, Leibniz Institute for Natural Product Research and Infection Biology – Hans Knöll Institute, Jena, Germany

^2^Faculty of Biological Sciences, Friedrich Schiller University Jena, Germany

^3^Center for Sepsis Control and Care (CSCC), Jena University Hospital, Jena, Germany

^4^Fungal Septomics, Septomics Research Center, Friedrich Schiller University and Leibniz Institute for Natural Product Research and Infection Biology – Hans Knöll Institute, Jena, Germany

^5^Institute for Hygiene and Microbiology, University of Würzburg, Würzburg, Germany

*** Correspondence:**Marc Thilo Figge
[thilo.figge@leibniz-hki.de](mailto:thilo.figge@leibniz-hki.de)

# Supplementary Figures and Tables

## Supplementary Figures
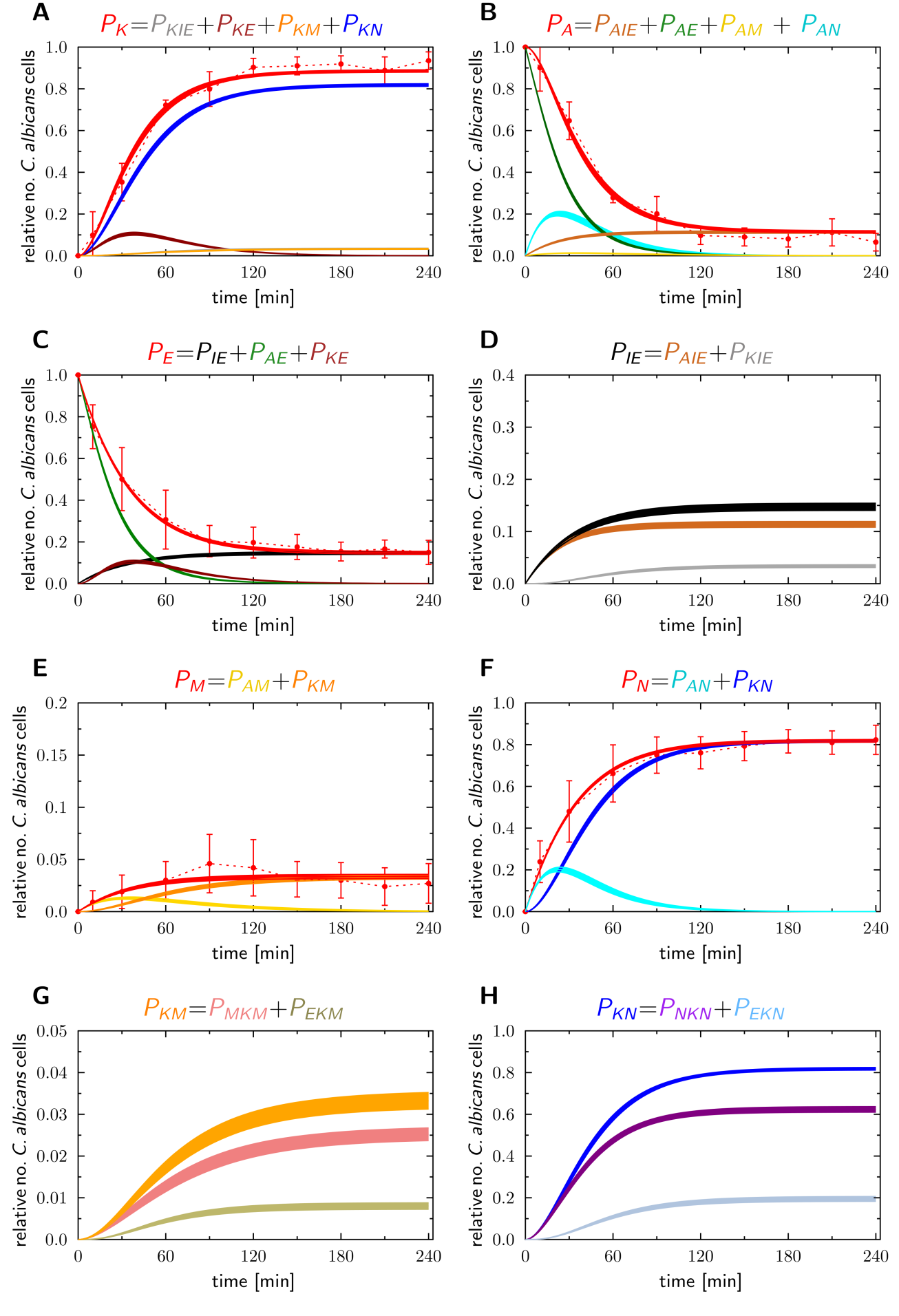


Supplementary Figure 1 Dynamics of combined units simulated by the SBM (red solid lines) with minimal least-squares error (LSE) to the experimental data from whole-blood infection assays with *C. albicans* (red dashed lines as guide for the eye). The error bars correspond to the standard deviations of five independent experiments. The thickness of the solid lines represents the standard deviation of the simulation results as obtained by 30 simulations for uniformly distributed transition rates as given in Table S1. The colored continuous lines represent the *in silico* kinetics of the fungal states that are referred to as the symbol with the same color. $\mathbf{(A)}$ The dynamics of killed fungal cells that were experimentally measured by survival assays. The corresponding combined unit $\boldsymbol{P}_{\boldsymbol{K}}$ comprises the states of fungal cells that are killed and immune-evasive ($\boldsymbol{P}_{\boldsymbol{KIE}}$), killed and extracellular ($\boldsymbol{P}_{\boldsymbol{KE}}$), killed in monocytes ($\boldsymbol{P}_{\boldsymbol{KM}}\boldsymbol{)}$ and killed in neutrophils ($\boldsymbol{P}_{\boldsymbol{KN}}$). $\mathbf{(B)}$ Time course of alive fungal cells that were measured by survival assays and simulated by the combined unit $\boldsymbol{P}_{\boldsymbol{A}}$. This is calculated by the sum of all states representing alive fungal cells, *i.e.* fungal states that are alive and immune-evasive ($\boldsymbol{P}_{\boldsymbol{AIE}}$), alive and extracellular ($\boldsymbol{P}_{\boldsymbol{AE}}$), and alive and in monocytes ($\boldsymbol{P}_{\boldsymbol{AM}}$) or in neutrophils ($\boldsymbol{P}_{\boldsymbol{AN}}$). $\mathbf{(C)}$ Kinetics of extracellular fungal cells that were measured by FACS analysis. These are compared with the dynamics of the combined unit $\boldsymbol{P}_{\boldsymbol{E}}$ that comprises states of fungal cells that are extracellular and alive ($\boldsymbol{P}_{\boldsymbol{AE}}$), extracellularly killed ($\boldsymbol{P}_{\boldsymbol{KE}}$) and immune-evasive ($\boldsymbol{P}_{\boldsymbol{IE}}$). $\mathbf{(D)}$ The *in silico* dynamics of fungal cells that are immune-evasive ($\boldsymbol{P}_{\boldsymbol{IE}}$) are the sum of alive ($\boldsymbol{P}_{\boldsymbol{AIE}}\boldsymbol{)}$ and killed ($\boldsymbol{P}_{\boldsymbol{KIE}}$) immune-evasive fungal cells at each time point of the simulation time. $\mathbf{(E)}$ The relative number of fungal cells in monocytes are experimentally measured by FACS analysis and simulated by the combined unit $\boldsymbol{P}_{\boldsymbol{M}}$ that is defined by the sum of fungal states in monocytes that are alive or killed, *i.e.* $\boldsymbol{P}_{\boldsymbol{AM}}$, $\boldsymbol{P}_{\boldsymbol{KM}}$, respectively. $\mathbf{(F)}$ The kinetics of fungal cells in neutrophils were measured using FACS analysis and are comparable with *in silico* dynamics of the combined unit $\boldsymbol{P}_{\boldsymbol{N}}$. This is defined as the sum of all fungal states that are in neutrophils and either alive ($\boldsymbol{P}_{\boldsymbol{AN}}$) or killed ($\boldsymbol{P}_{\boldsymbol{KN}}$). $\mathbf{(G)}$ *In silico* kinetics of killed fungal cells in monocytes ($\boldsymbol{P}_{\boldsymbol{KM}}$) that are defined as the sum of intracellularly killed ($\boldsymbol{P}_{\boldsymbol{MKM}}$) and extracellularly killed ($\boldsymbol{P}_{\boldsymbol{EKM}}$) pathogens at each simulation time point. $\mathbf{(H)}$ *In silico* time course of fungal cells in neutrophils that were killed ($\boldsymbol{P}_{\boldsymbol{KN}}$). These cells were either killed intracellularly in neutrophils ($\boldsymbol{P}_{\boldsymbol{NKN}}$) or killed extracellularly ($\boldsymbol{P}_{\boldsymbol{EKN}}$).


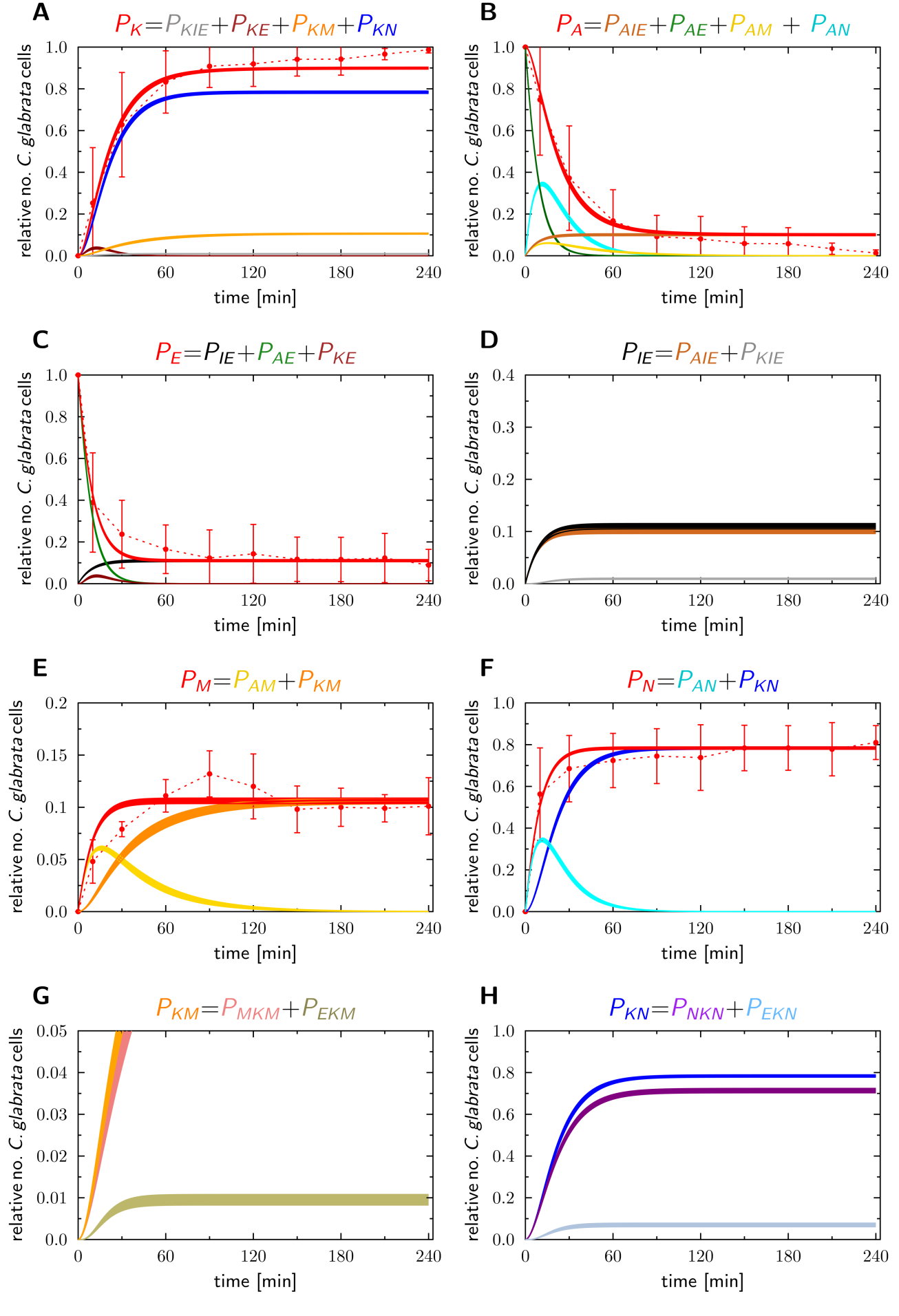


Supplementary Figure 2 Dynamics of combined units simulated by the SBM (red solid lines) with minimal least-squares error (LSE) to the experimental data from whole-blood infection assays with *C. glabrata* (red dashed lines as guide for the eye). The error bars correspond to the standard deviations of five independent experiments. The thickness of the solid lines represents the standard deviation of the simulation results as obtained by 30 simulations for uniformly distributed transition rates as given in Table S1. The colored continuous lines represent the *in silico* kinetics of the fungal states that are referred to as the symbol with the same color. $\mathbf{(A)}$ The dynamics of killed fungal cells that were experimentally measured by survival assays. The corresponding combined unit $\boldsymbol{P}_{\boldsymbol{K}}$ comprises the states of fungal cells that are killed and immune-evasive ($\boldsymbol{P}_{\boldsymbol{KIE}}$), killed and extracellular ($\boldsymbol{P}_{\boldsymbol{KE}}$), killed in monocytes ($\boldsymbol{P}_{\boldsymbol{KM}}\boldsymbol{)}$ and killed in neutrophils ($\boldsymbol{P}_{\boldsymbol{KN}}$). $\mathbf{(B)}$ Time course of alive fungal cells that were measured by survival assays and simulated by the combined unit $\boldsymbol{P}_{\boldsymbol{A}}$. This is calculated by the sum of all states representing alive fungal cells, *i.e.* fungal states that are alive and immune-evasive ($\boldsymbol{P}_{\boldsymbol{AIE}}$), alive and extracellular ($\boldsymbol{P}_{\boldsymbol{AE}}$), and alive and in monocytes ($\boldsymbol{P}_{\boldsymbol{AM}}$) or in neutrophils ($\boldsymbol{P}_{\boldsymbol{AN}}$). $\mathbf{(C)}$ Kinetics of extracellular fungal cells that were measured by FACS analysis. These are compared with the dynamics of the combined unit $\boldsymbol{P}_{\boldsymbol{E}}$ that comprises states of fungal cells that are extracellular and alive ($\boldsymbol{P}_{\boldsymbol{AE}}$), extracellularly killed ($\boldsymbol{P}_{\boldsymbol{KE}}$) and immune-evasive ($\boldsymbol{P}_{\boldsymbol{IE}}$). $\mathbf{(D)}$ The *in silico* dynamics of fungal cells that are immune-evasive ($\boldsymbol{P}_{\boldsymbol{IE}}$) are the sum of alive ($\boldsymbol{P}_{\boldsymbol{AIE}}\boldsymbol{)}$ and killed ($\boldsymbol{P}_{\boldsymbol{KIE}}$) immune-evasive fungal cells at each time point of the simulation time. $\mathbf{(E)}$ The relative number of fungal cells in monocytes are experimentally measured by FACS analysis and simulated by the combined unit $\boldsymbol{P}_{\boldsymbol{M}}$ that is defined by the sum of fungal states in monocytes that are alive or killed, *i.e.* $\boldsymbol{P}_{\boldsymbol{AM}}$, $\boldsymbol{P}_{\boldsymbol{KM}}$, respectively. $\mathbf{(F)}$ The kinetics of fungal cells in neutrophils were measured using FACS analysis and are comparable with *in silico* dynamics of the combined unit $\boldsymbol{P}_{\boldsymbol{N}}$. This is defined as the sum of all fungal states that are in neutrophils and either alive ($\boldsymbol{P}_{\boldsymbol{AN}}$) or killed ($\boldsymbol{P}_{\boldsymbol{KN}}$). $\mathbf{(G)}$ *In silico* kinetics of killed fungal cells in monocytes ($\boldsymbol{P}_{\boldsymbol{KM}}$) that are defined as the sum of intracellularly killed ($\boldsymbol{P}_{\boldsymbol{MKM}}$) and extracellularly killed ($\boldsymbol{P}_{\boldsymbol{EKM}}$) pathogens at each simulation time point. $\mathbf{(H)}$ *In silico* time course of fungal cells in neutrophils that were killed ($\boldsymbol{P}_{\boldsymbol{KN}}$). These cells were either killed intracellularly in neutrophils ($\boldsymbol{P}_{\boldsymbol{NKN}}$) or killed extracellularly ($\boldsymbol{P}_{\boldsymbol{EKN}}$).

**
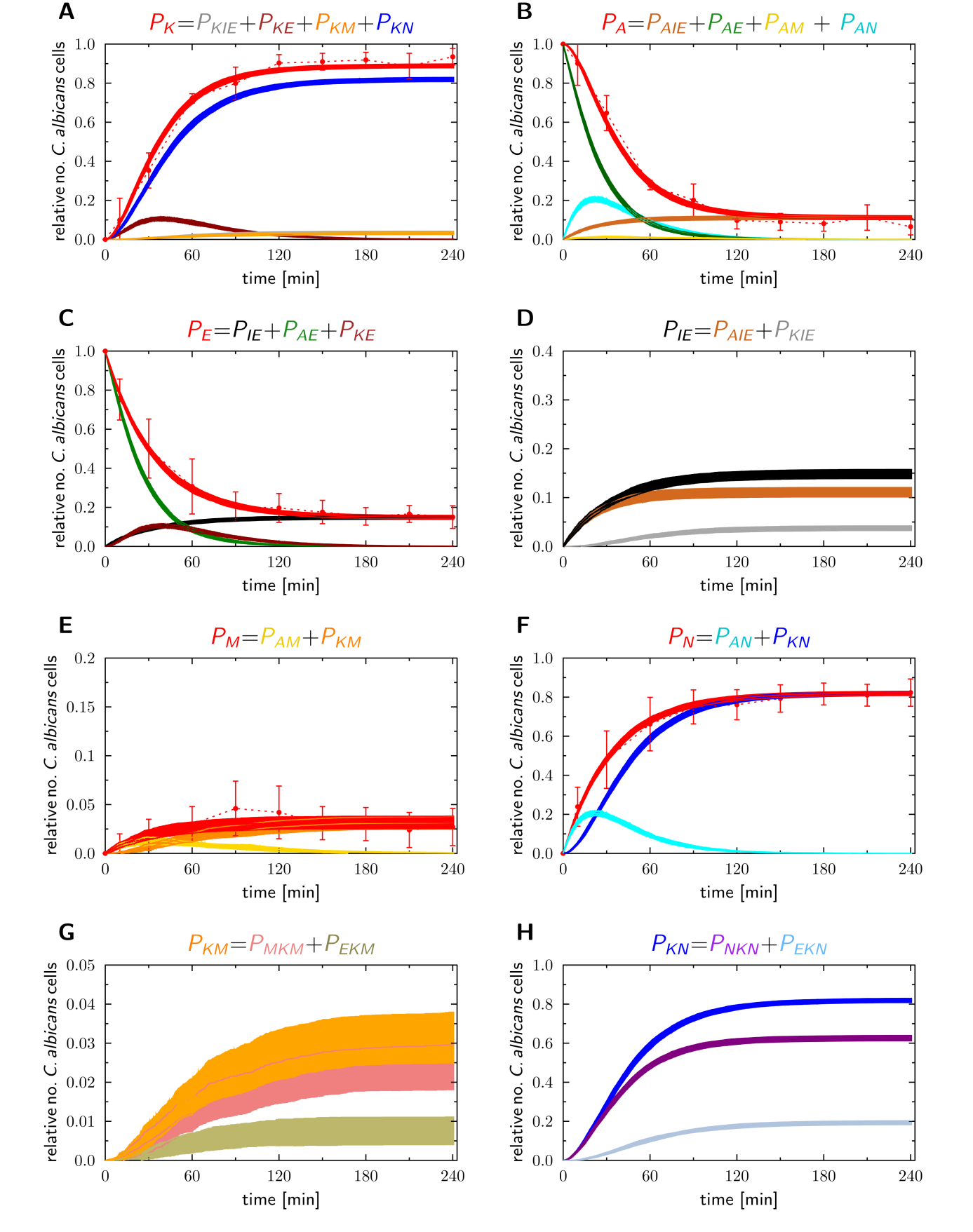
**

Supplementary Figure 3 Dynamics of combined units simulated by the ABM (red solid lines) with minimal least-squares error (LSE) to the experimental data from whole-blood infection assays with *C. albicans* (red dashed lines as guide for the eye). The error bars correspond to the standard deviations of five independent experiments. The thickness of the solid lines represents the standard deviation of the simulation results as obtained by 30 simulations for uniformly distributed transition rates as given in Table S1. The colored continuous lines represent the *in silico* kinetics of the fungal states that are referred to as the symbol with the same color. $\mathbf{(A)}$ The dynamics of killed fungal cells that were experimentally measured by survival assays. The corresponding combined unit $\boldsymbol{P}_{\boldsymbol{K}}$ comprises the states of fungal cells that are killed and immune-evasive ($\boldsymbol{P}_{\boldsymbol{KIE}}$), killed and extracellular ($\boldsymbol{P}_{\boldsymbol{KE}}$), killed in monocytes ($\boldsymbol{P}_{\boldsymbol{KM}}\boldsymbol{)}$ and killed in neutrophils ($\boldsymbol{P}_{\boldsymbol{KN}}$). $\mathbf{(B)}$ Time course of alive fungal cells that were measured by survival assays and simulated by the combined unit $\boldsymbol{P}_{\boldsymbol{A}}$. This is calculated by the sum of all states representing alive fungal cells, *i.e.* fungal states that are alive and immune-evasive ($\boldsymbol{P}_{\boldsymbol{AIE}}$), alive and extracellular ($\boldsymbol{P}_{\boldsymbol{AE}}$), and alive and in monocytes ($\boldsymbol{P}_{\boldsymbol{AM}}$) or in neutrophils ($\boldsymbol{P}_{\boldsymbol{AN}}$). $\mathbf{(C)}$ Kinetics of extracellular fungal cells that were measured by FACS analysis. These are compared with the dynamics of the combined unit $\boldsymbol{P}_{\boldsymbol{E}}$ that comprises states of fungal cells that are extracellular and alive ($\boldsymbol{P}_{\boldsymbol{AE}}$), extracellularly killed ($\boldsymbol{P}_{\boldsymbol{KE}}$) and immune-evasive ($\boldsymbol{P}_{\boldsymbol{IE}}$). $\mathbf{(D)}$ The *in silico* dynamics of fungal cells that are immune-evasive ($\boldsymbol{P}_{\boldsymbol{IE}}$) are the sum of alive ($\boldsymbol{P}_{\boldsymbol{AIE}}\boldsymbol{)}$ and killed ($\boldsymbol{P}_{\boldsymbol{KIE}}$) immune-evasive fungal cells at each time point of the simulation time. $\mathbf{(E)}$ The relative number of fungal cells in monocytes are experimentally measured by FACS analysis and simulated by the combined unit $\boldsymbol{P}_{\boldsymbol{M}}$ that is defined by the sum of fungal states in monocytes that are alive or killed, *i.e.* $\boldsymbol{P}_{\boldsymbol{AM}}$, $\boldsymbol{P}_{\boldsymbol{KM}}$, respectively. $\mathbf{(F)}$ The kinetics of fungal cells in neutrophils were measured using FACS analysis and are comparable with *in silico* dynamics of the combined unit $\boldsymbol{P}_{\boldsymbol{N}}$. This is defined as the sum of all fungal states that are in neutrophils and either alive ($\boldsymbol{P}_{\boldsymbol{AN}}$) or killed ($\boldsymbol{P}_{\boldsymbol{KN}}$). $\mathbf{(G)}$ *In silico* kinetics of killed fungal cells in monocytes ($\boldsymbol{P}_{\boldsymbol{KM}}$) that are defined as the sum of intracellularly killed ($\boldsymbol{P}_{\boldsymbol{MKM}}$) and extracellularly killed ($\boldsymbol{P}_{\boldsymbol{EKM}}$) pathogens at each simulation time point. $\mathbf{(H)}$ *In silico* time course of fungal cells in neutrophils that were killed ($\boldsymbol{P}_{\boldsymbol{KN}}$). These cells were either killed intracellularly in neutrophils ($\boldsymbol{P}_{\boldsymbol{NKN}}$) or killed extracellularly ($\boldsymbol{P}_{\boldsymbol{EKN}}$).


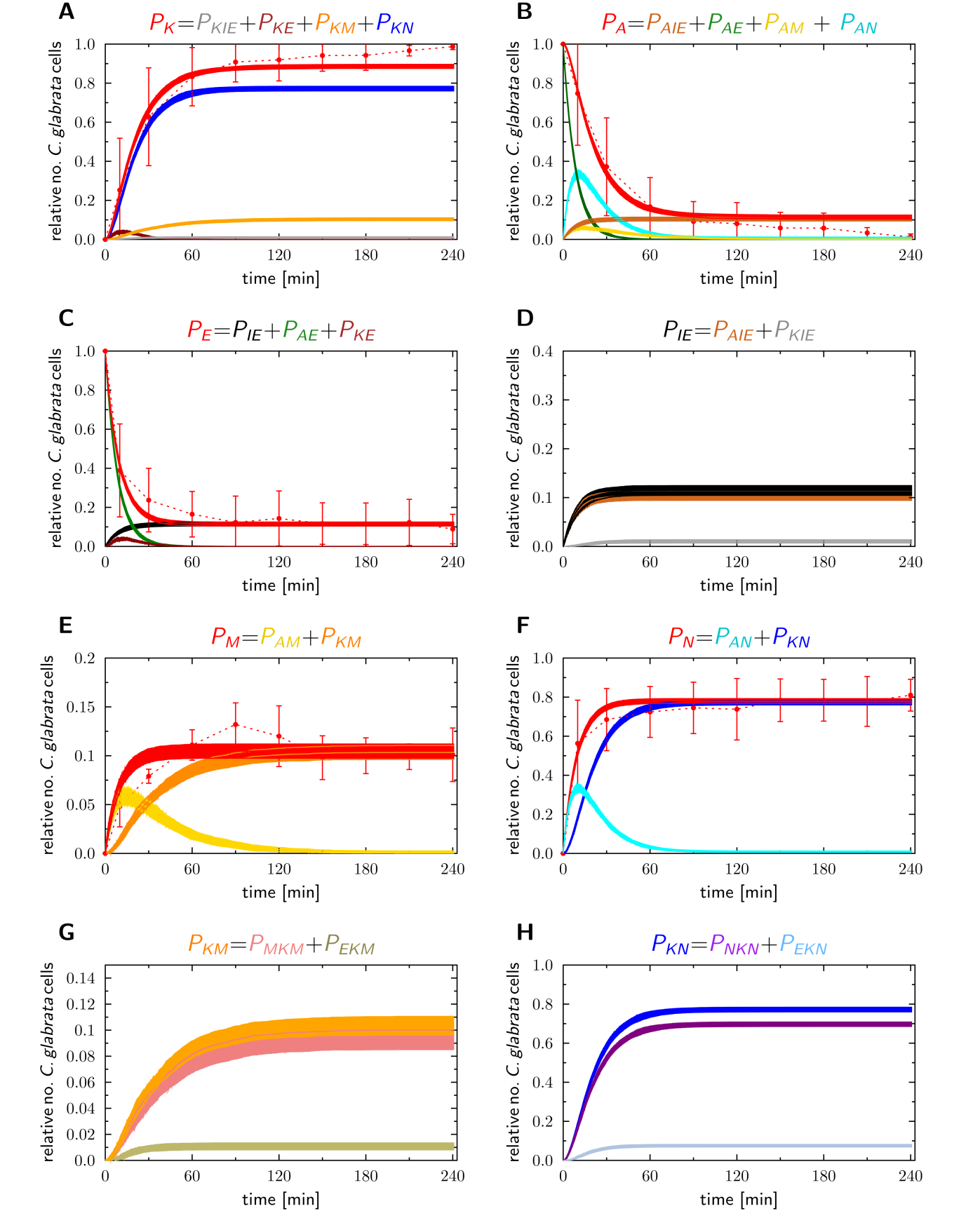


Supplementary Figure 4 Dynamics of combined units simulated by the ABM (red solid lines) with minimal least-squares error (LSE) to the experimental data from whole-blood infection assays with *C. glabrata* (red dashed lines as guide for the eye). The error bars correspond to the standard deviations of five independent experiments. The thickness of the solid lines represents the standard deviation of the simulation results as obtained by 30 simulations for uniformly distributed transition rates as given in Table S1. The colored continuous lines represent the *in silico* kinetics of the fungal states that are referred to as the symbol with the same color. $\mathbf{(A)}$ The dynamics of killed fungal cells that were experimentally measured by survival assays. The corresponding combined unit $\boldsymbol{P}_{\boldsymbol{K}}$ comprises the states of fungal cells that are killed and immune-evasive ($\boldsymbol{P}_{\boldsymbol{KIE}}$), killed and extracellular ($\boldsymbol{P}_{\boldsymbol{KE}}$), killed in monocytes ($\boldsymbol{P}_{\boldsymbol{KM}}\boldsymbol{)}$ and killed in neutrophils ($\boldsymbol{P}_{\boldsymbol{KN}}$). $\mathbf{(B)}$ Time course of alive fungal cells that were measured by survival assays and simulated by the combined unit $\boldsymbol{P}_{\boldsymbol{A}}$. This is calculated by the sum of all states representing alive fungal cells, *i.e.* fungal states that are alive and immune-evasive ($\boldsymbol{P}_{\boldsymbol{AIE}}$), alive and extracellular ($\boldsymbol{P}_{\boldsymbol{AE}}$), and alive and in monocytes ($\boldsymbol{P}_{\boldsymbol{AM}}$) or in neutrophils ($\boldsymbol{P}_{\boldsymbol{AN}}$). $\mathbf{(C)}$ Kinetics of extracellular fungal cells that were measured by FACS analysis. These are compared with the dynamics of the combined unit $\boldsymbol{P}_{\boldsymbol{E}}$ that comprises states of fungal cells that are extracellular and alive ($\boldsymbol{P}_{\boldsymbol{AE}}$), extracellularly killed ($\boldsymbol{P}_{\boldsymbol{KE}}$) and immune-evasive ($\boldsymbol{P}_{\boldsymbol{IE}}$). $\mathbf{(D)}$ The *in silico* dynamics of fungal cells that are immune-evasive ($\boldsymbol{P}_{\boldsymbol{IE}}$) are the sum of alive ($\boldsymbol{P}_{\boldsymbol{AIE}}\boldsymbol{)}$ and killed ($\boldsymbol{P}_{\boldsymbol{KIE}}$) immune-evasive fungal cells at each time point of the simulation time. $\mathbf{(E)}$ The relative number of fungal cells in monocytes are experimentally measured by FACS analysis and simulated by the combined unit $\boldsymbol{P}_{\boldsymbol{M}}$ that is defined by the sum of fungal states in monocytes that are alive or killed, *i.e.* $\boldsymbol{P}_{\boldsymbol{AM}}$, $\boldsymbol{P}_{\boldsymbol{KM}}$, respectively. $\mathbf{(F)}$ The kinetics of fungal cells in neutrophils were measured using FACS analysis and are comparable with *in silico* dynamics of the combined unit $\boldsymbol{P}_{\boldsymbol{N}}$. This is defined as the sum of all fungal states that are in neutrophils and either alive ($\boldsymbol{P}_{\boldsymbol{AN}}$) or killed ($\boldsymbol{P}_{\boldsymbol{KN}}$). $\mathbf{(G)}$ *In silico* kinetics of killed fungal cells in monocytes ($\boldsymbol{P}_{\boldsymbol{KM}}$) that are defined as the sum of intracellularly killed ($\boldsymbol{P}_{\boldsymbol{MKM}}$) and extracellularly killed ($\boldsymbol{P}_{\boldsymbol{EKM}}$) pathogens at each simulation time point. $\mathbf{(H)}$ *In silico* time course of fungal cells in neutrophils that were killed ($\boldsymbol{P}_{\boldsymbol{KN}}$). These cells were either killed intracellularly in neutrophils ($\boldsymbol{P}_{\boldsymbol{NKN}}$) or killed extracellularly ($\boldsymbol{P}_{\boldsymbol{EKN}}$).

**
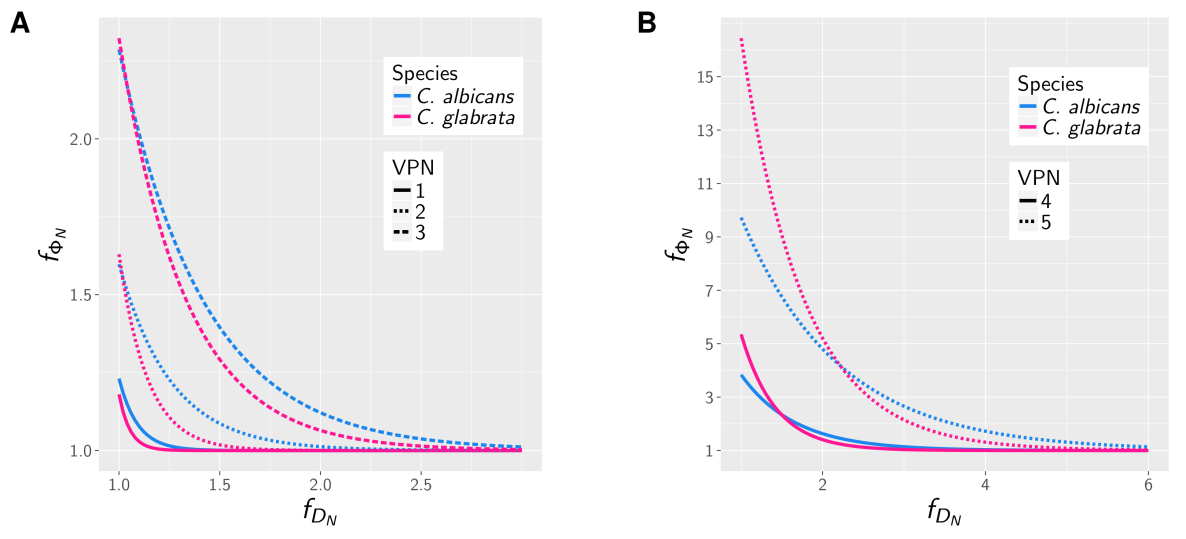
**

Supplementary Figure 5 The fitted curves $\boldsymbol{f}_{\boldsymbol{\phi}_{\boldsymbol{N}}}\boldsymbol{=1+a\cdot}\boldsymbol{e}^{\boldsymbol{-b\cdot}\boldsymbol{f}_{\boldsymbol{D}_{\boldsymbol{N}}}}$ at the transition to the non-neutropenic infection outcome with *C. albicans* (blue) or *C. glabrata* (pink) for VNP-1 – 3 $\mathbf{(A)}$ and VNP-4 and VNP-5 $\mathbf{(B)}$.

## Supplementary Tables

Supplementary Table 1 Immune Reaction Rates estimated by fitting the state-based model (SBM) to the experimental data. The SBM comprises seven rates: the phagocytosis rate for neutrophils ($\boldsymbol{\Phi}_{\boldsymbol{N}}$) and for monocytes ($\boldsymbol{\Phi}_{\boldsymbol{M}}$), the rate for intracellular killing by neutrophils ($\boldsymbol{\kappa}_{\boldsymbol{N}}$) and by monocytes ($\boldsymbol{\kappa}_{\boldsymbol{M}}$), the rate for immune evasion by the pathogens ($\boldsymbol{\rho}$), the rates that define the extracellular killing by antimicrobial peptides, *i.e.* $\boldsymbol{\gamma}$ and ${\bar{\boldsymbol{\kappa}}}_{\boldsymbol{EK}}$.$\boldsymbol{\gamma}$).

| Rate | *C. albicans* | | | *C. glabrata* | | |
| --- | --- | --- | --- | --- | --- | --- |
|  | mean ${10}^{-2}{min}^{-1}$ | sd  ${10}^{-3} {min}^{-1}$ | sd  $[\%]$ | mean ${10}^{-2}{min}^{-1}$ | sd  ${10}^{-3} {min}^{-1}$ | sd  $[\%]$ |
| $\boldsymbol{\Phi}_{\boldsymbol{N}}$ | $2.966$ | $0.611$ | $2.06$ | $10.11$ | $1.953$ | $1.93$ |
| $\boldsymbol{\Phi}_{\boldsymbol{M}}$ | $1.228$ | $0.746$ | $6.07$ | $13.69$ | $4.003$ | $2.92$ |
| $\boldsymbol{\kappa}_{\boldsymbol{N}}$ | $5.319$ | $4.868$ | $9.15$ | $6.98$ | $4.959$ | $7.11$ |
| $\boldsymbol{\kappa}_{\boldsymbol{M}}$ | $2.098$ | $3.056$ | $14.57$ | $3.219$ | $3.433$ | $10.66$ |
| $\boldsymbol{\rho}$ | $0.439$ | $0.211$ | $4.81$ | $1.173$ | $0.481$ | $4.1$ |
| $\boldsymbol{\gamma}$ | $2.129$ | $1.868$ | $8.77$ | $5.389$ | $4.028$ | $7.47$ |
| ${\bar{\boldsymbol{\kappa}}}_{\boldsymbol{EK}}$ | $21.78$ | $17.46$ | $8.02$ | $22.98$ | $27.74$ | $12.07$ |

Supplementary Table 2 Values for killed cells ($\boldsymbol{P}_{\boldsymbol{K}}$) as well as alive and immune-evasive cells ($\boldsymbol{P}_{\boldsymbol{AIE}}$) at the transitions between different severity degrees of neutropenia for *C. albicans* and *C. glabrata* infection at four hours post infection. These values are used as a pattern for classification of the outcome of *in silico* treatment of virtual neutropenic patients.

|  |  | mild | moderate | severe |
| --- | --- | --- | --- | --- |
| *C. albicans* | $P_{K}$ | $0.713\pm0.014$ | $0.623\pm0.017$ | $0.464\pm0.02$ |
|  | $P_{AIE}$ | $0.26\pm0.015$ | $0.322\pm0.016$ | $0.417\pm0.016$ |
| *C. glabrata* | $P_{K}$ | $0.757\pm0.012$ | $0.707\pm0.015$ | $0.612\pm0.018$ |
|  | $P_{AIE}$ | $0.236\pm0.012$ | $0.286\pm0.015$ | $0.38\pm0.017$ |

Supplementary Table 3 *In silico* treatment of VNP in infection with *C. albicans* and *C. glabrata* is predicted to reach the transition to the non-neutropenic infection outcome for VNP with various severity degrees of neutropenia. The function $\boldsymbol{f}_{\boldsymbol{\phi}_{\boldsymbol{N}}}\boldsymbol{=1+a\cdot}\boldsymbol{e}^{\boldsymbol{-b\cdot}\boldsymbol{f}_{\boldsymbol{D}_{\boldsymbol{N}}}}$ is fitted to this transition with the parameters $\boldsymbol{a}$ and $\boldsymbol{b}$.

| Virtual Neutropenic Patient Type (VNP) | *C. albicans* | | *C. glabrata* | |
| --- | --- | --- | --- | --- |
|  | a | b | a | b |
| $\boldsymbol{1}$ | $12637.28$ | $10.91$ | $16974577.77$ | $18.36$ |
| $\boldsymbol{2}$ | $28.37$ | $3.86$ | $755.76$ | $7.09$ |
| $\boldsymbol{3}$ | $13.63$ | $2.36$ | $27.11$ | $3.02$ |
| $\boldsymbol{4}$ | $12.71$ | $1.5$ | $46.56$ | $2.37$ |
| $\boldsymbol{5}$ | $20$ | $0.83$ | $56.66$ | $1.3$ |
